# The Strategy of Boosting the Immune System Under the COVID-19 Pandemic

**DOI:** 10.3389/fvets.2020.570748

**Published:** 2021-01-08

**Authors:** Mahmoud Alagawany, Youssef A. Attia, Mayada R. Farag, Shaaban S. Elnesr, Sameer A. Nagadi, Manal E. Shafi, Asmaa F. Khafaga, Husein Ohran, Abdulaziz A. Alaqil, Mohamed E. Abd El-Hack

**Affiliations:** ^1^Department of Poultry, Faculty of Agriculture, Zagazig University, Zagazig, Egypt; ^2^Agriculture Department, Faculty of Environmental Sciences, King Abdulaziz University, Jeddah, Saudi Arabia; ^3^The Strategic Center to Kingdom Vision Realization, King Abdulaziz University, Jeddah, Saudi Arabia; ^4^Animal and Poultry Production Department, Faculty of Agriculture, Damanhour University, Damanhour, Egypt; ^5^Forensic Medicine and Toxicology Department, Faculty of Veterinary Medicine, Zagazig University, Zagazig, Egypt; ^6^Department of Poultry Production, Faculty of Agriculture, Fayoum University, Fayoum, Egypt; ^7^Department of Biological Sciences, Zoology, King Abdulaziz University, Jeddah, Saudi Arabia; ^8^Department of Pathology, Faculty of Veterinary Medicine, Alexandria University, Alexandria, Egypt; ^9^Department of Physiology, Veterinary Faculty, University of Sarajevo, Sarajevo, Bosnia and Herzegovina; ^10^Department of Animal and Fish Production, King Faisal University, Al-Hufof, Saudi Arabia

**Keywords:** COVID-19, SARS—CoV-2, vitamins, minerals, probiotics

## Abstract

The novel coronavirus (SARS-CoV-2) infection (COVID-19) has raised considerable concern on the entire planet. On March 11, 2020, COVID-19 was categorized by the World Health Organization (WHO) as a pandemic infection, and by March 18, 2020, it has spread to 146 countries. The first internal defense line against numerous diseases is personalized immunity. Although it cannot be claimed that personalized nutrition will have an immediate impact on a global pandemic, as the nutritional interventions required a long time to induce beneficial outcomes on immunity development, nutritional strategies are still able to clarify and have a beneficial influence on the interplay between physiology and diet, which could make a positive contribution to the condition in the next period. As such, a specific goal for every practitioner is to evaluate different tests to perceive the status of the patient, such as markers of inflammation, insulin regulation, and nutrient status, and to detect possible imbalances or deficiencies. During the process of disease development, the supplementation and addition of different nutrients and nutraceuticals can influence not only the viral replication but also the cellular mechanisms. It is essential to understand that every patient has its individual needs. Even though many nutrients, nutraceuticals, and drugs have beneficial effects on the immune response and can prevent or ameliorate viral infections, it is essential to detect at what stage in COVID-19 progression the patient is at the moment and decide what kind of nutrition intervention is necessary. Furthermore, understanding the pathogenesis of coronavirus infection is critical to make proper recommendations.

## Introduction

Coronaviridae is one of the families of viruses that are known to cause diseases in birds and mammals ranging from the common cold to severe acute respiratory syndrome (SRAS). A new species that belongs to this family emerged in late 2019, causing an outbreak of a respiratory disease in the eastern part of Asia. The first species of coronavirus infecting humans (229E) was observed in 1931 and isolated first in 1965. Ultimately, the virus has evolved into the novel coronavirus (2019-n-CoV).

Regarding the current situation of the COVID-19 pandemic, in which there is no optimal vaccination or cure, the functionality and efficiency of the immune response is the key factor in the defense against viral infections. Several nutrients, especially vitamins and microelements, are crucial for the immune system to function normally (1). Moreover, dietary supplementations of such nutrients have beneficial impacts on the immune responses to viral infections. Studies showed that after influenza vaccination, the supplementation of vitamins A and D raised the humoral immunity of pediatric patients (2). In patients with torque teno virus (TTV), dietary supplementation with high levels of zinc resulted in improved immunity (3). Also, high doses of selenium had positive effects on the immune response after influenza vaccination (4).

In the literature, several micronutrients, herbal therapeutics, and probiotics were reported to have positive effects in both treatment and prophylaxis of viral infections (5). Also, various nutraceuticals were found to have immunomodulatory effects (6, 7). Malnutrition, as a major cause of increased morbidity and mortality, also has a remarkable economic influence on the healthcare system, depending on the economic situations of each country (8). Such repercussions of malnutrition are reflections of the increased infection rates and delayed recovery as well. Also, in infection, the requirements for various nutrients become higher (9). Nutrition is an essential regulator of the immune homeostasis, and even small deficiencies of some micronutrients could disturb the immune response (10). Lately, Calder et al. reported that balanced nutrition is of crucial importance in the protection from viral infection (11), while Jayawardena et al. recommended that some nutraceuticals and probiotics could be useful in possible prevention and management of COVID-19 (12).

Regarding the COVID-19, it is fundamental to demonstrate the data on increasing the immune response to viral infections. The present review paper principally subjects influenza-like viral infections. Nonetheless, other viral infections have also been taken into consideration. Therefore, practical recommendations on using nutrients for the prevention and therapy of COVID-19 are given. Accordingly, the aim of this review was to provide a complementary overview of the recently available scientific literature on the strategy of using nutraceuticals in boosting the immune system under the COVID-19 pandemic. Emphasis is placed on the supplementations of nutraceuticals, probiotics, vitamins, and trace elements.

## Symptoms and Risk Factors of COVID-19

Two to 14 days after exposure to the virus, the usual clinical signs are dry cough and shortness of breath. The Centers for Disease Control and Prevention (CDC) reported that the appearance of fever, chills, myalgia, headache, as well as the loss of smell and taste, could indicate an infection. The majority of humans have mild symptoms if any occur, and it has been reported that ca. 80% of infected people do not need hospitalization (13).

Nevertheless, in some individuals, the infection can cause severe problems. With aging, the immune response becomes weaker (immunosenescence), and the levels of inflammatory mediators in the blood increases (inflammageing). The high-risk group of COVID-19 that are clinically vulnerable includes primarily elderly individuals and those with inflammation-associated conditions such as overweight and obesity, chronic obstructive pulmonary disease, cardiovascular diseases, diabetes, kidney disease, etc. The majority of severe COVID-19 cases and mortalities were within the abovementioned group of individuals, although it is worrisome that younger and healthy individuals are showing up in this cohort (14, 15).

**Figure 1:**
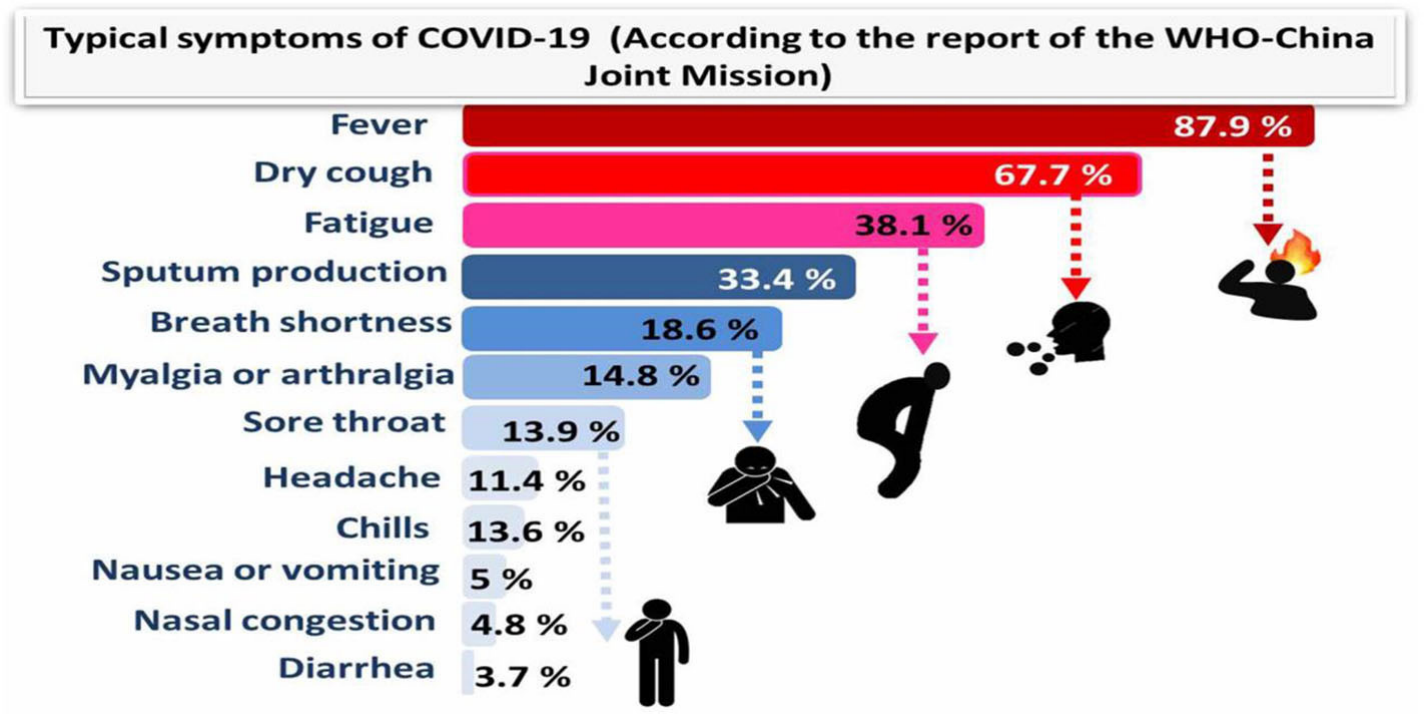
Typical symptoms of COVID-19 (according to the report of the WHO-China Joint Mission) relied on 55,924 COVID-19 confirmed cases (February 2020).

The virus rapidly multiplies and infects the surrounding cells throughout the respiratory system. At the time the virus gets to the lungs, an inflammatory process starts in the mucous membranes and damages the alveoli, which have difficulties in supplying oxygen, resulting in breathing difficulties. This can cause swelling in the lungs, which can result in the accumulation of fluids and dead cells and, finally, severe pneumonia. The infection can spread through mucous membranes of the body, such as the digestive system. In some cases, gastrointestinal symptoms like diarrhea, indigestion, and vomiting have been reported along with respiratory symptoms, and studies propose the possibility of fecal–oral transmission (16). The typical symptoms of COVID-19 and their incidence percentage in 55,924 laboratory confirmed cases are illustrated in Figure 1 (according to the report of the WHO-China Joint Mission; 20 Feb., 2020).

SARS-CoV-2 has been isolated from the brainstems of positive patients, which indicates that this virus has common pathways of transmission with other coronaviruses that can spread through synapse-connected routes from the lung and airways. The virus can cause problems in different parts of the body, such as the heart, liver, and kidneys, particularly in the later stage of the disease. Fatal cases of COVID-19 can be caused by multiple organ failure, especially in individuals with genetic immune factors (17, 18).

## The Role of Nutraceuticals in Raising the Immunity for Confrontation of the COVID-19 Pandemic

This review highlights the nutritional interventions to increase the immune response in the body during viral infections, especially considering the novel coronavirus pandemic. Due to their beneficial effects on general healthcare and disease prophylaxis, nutraceuticals have been gaining more and more importance lately (19). Nutraceutical is composed of two words: nutrient and pharmaceutical. It has a vital role in maintaining the healthy body and thus prevents the body from diseases. Several nutraceuticals have been reported to have a significant impact on enhancing the immune system and aid in the treatment and/or protection of viral infections, particularly influenza-like diseases (19, 20). In the current COVID-19 pandemic situation and unavailability of the drugs, these safe alternatives such as nutraceuticals can provide a significant value against the combat of COVID-19.

Over an extended period, obesity has been correlated with a higher occurrence of chronic diseases. Research findings indicate that obesity increases infectious diseases risk (21). The latest evidences in patients suffering from COVID-19 reported that the occurrence and severity of the disease are related to BMI ≥ 28 kg m^−2^ (22). Based on translational data, it could be a result of metabolic alterations.

In obese patients, the profile of T cells showed inhibition of the function and activation of these crucial immune cells (23). El-Kader and Al-Jiffri (24) conducted a study on 100 obese individuals with chronic infection of HIV and demonstrated that leukocytes, monocytes, total count of neutrophils, CD3, CD4, and CD8 lymphocytes showed significantly lower mean values in the individuals that went through a weight loss program, compared with the control group (24). However, there are two apparent limitations of this review. Firstly, due to the heterogenic nature of the studies, a meta-analysis was not carried out. Secondly, the review did not include studies on the impact of nutrient supplementation in hepatitis C virus (HCV) patients. Despite this, we think that clinical trials on HCV may enrich the aim of this review, discussing the respiratory infections such as COVID-19.

Qualitative evidences by the Jadad scale identified trials (<30%) with a score of <3 points, showing low qualities of methodology. Such studies were not ignored, mainly since a meta-analysis was not done for pooled estimates. On the other side, 24 types of research (>54%) had a score >3, which suggests a valuable methodology. Although it is well-known that exercise is a very active booster of immunity (25), whereas stress strongly alters the immune response (26), this review did not consider such studies as they are outside the scope of this paper.

Several *in vivo* and *in vitro* researches on animals have investigated the antiviral properties of trace elements, vitamins, and other nutraceuticals (27, 28). Nevertheless, it is not easy to give conclusions or make recommendations from these studies, and there is a need for further human clinical trials regarding COVID-19.

Micronutrient deficiencies have to be determined in the early stages to set the right therapeutic dose. If the individual micronutrient deficiencies are absent, each malnourished individual should be provided with a multivitamin and mineral (MVM) supplement (29). To increase the immune response of the body, an obese individual (BMI > 25 kg m^−2^) should reduce at least 5% of the weight of the body (30). Diabetes mellitus patients must have a balanced food to keep normal glucose levels and increase immunity (31) by having diets with the low glycemic index, limiting the consumptions of high fat and sugary or starchy diets, and choosing lean protein varieties (31).

## The Role of Vitamins

Numerous vitamins are crucial for the normal functions of the immune response (1). For maintaining the vitamin homeostasis in the body, it is vital to have a varied and balanced diet (32). The dietary supplementation of vitamin D may have positive effects on individuals who are either insufficient or deficient. Evidence supporting the role of vitamin D in reducing the risk of COVID-19 includes the fact that the outbreak occurred in winter, a time when 25-hydroxyvitamin D (25(OH)D) concentrations are lowest; that the number of cases in the Southern Hemisphere near the end of summer are low; that vitamin D deficiency has been found to contribute to acute respiratory distress syndrome; and that case-fatality rates increase with age and with chronic disease comorbidity, both of which are associated with lower 25(OH)D concentration (33). Vitamin E, as a well-known antioxidant, also has functions in regulating the immune response. Meanwhile, numerous studies showed that supplementation with vitamin E could have harmful effects on the immune system, especially in cancer and cardiovascular diseases. There was no conclusive evidence of the role of vitamin E in the treatment of COVID-19, but it is believed that vitamin E protects the integrity of cell membranes from damage caused by free radicals and has the potential to influence both innate and adaptive immunity.

**Figure 2:**
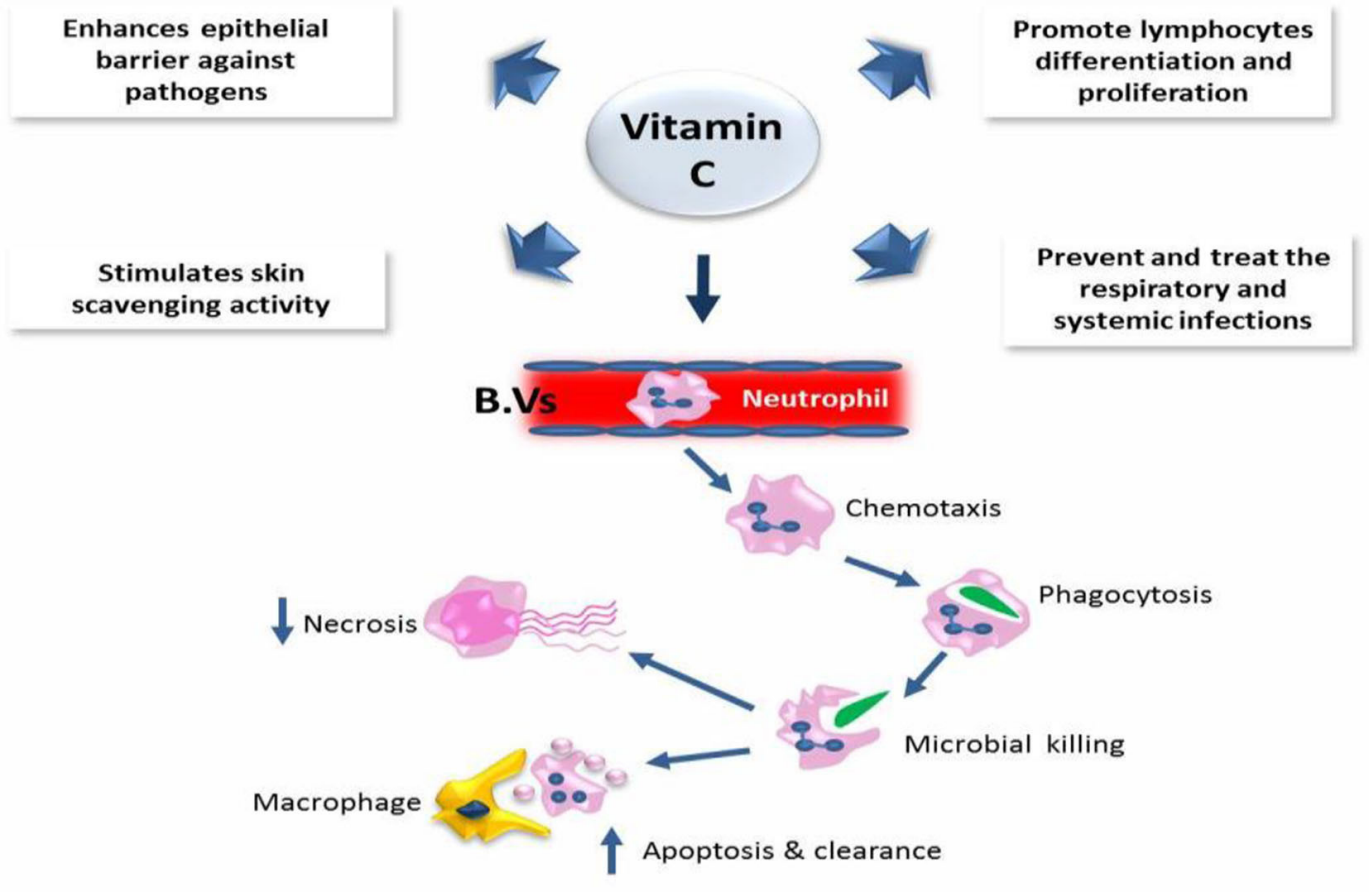
The role of vitamin C in the immune defense.

Moreover, excessive amounts of vitamin E could have fatal consequences. A recent study reported that dietary supplementation with high concentrations of micronutrients and vitamins C and D is an effective and low-cost method to intensify the immune response to COVID-19 and similar respiratory diseases (33, 34). It is well-known that vitamins C and D are essential for the immune system. Vitamin C takes part in the development and functionality of various immune cells and the production of antibodies. The contribution of vitamin C in immune response has been suggested due to the enhancement of different cellular functions of innate and adaptive immunity. Vitamin C enhances the function of epithelial barrier against pathogens and stimulates skin scavenging activity to protect against the environmental oxidative stress. In addition, it could accumulate in neutrophils to promote chemotaxis phagocytosis and with subsequent microbial killing. It is also required for apoptosis and neutrophil clearance from the infection sites, which resulted in a reduction of necrosis and possible tissue damage. In B and T lymphocytes, vitamin C might promote the cellular differentiation and proliferation due to its gene-regulating activities. Therefore, the deficiency of vitamin C may result in immunity impairment and increased susceptibility to infections. Therefore, infections may have a significant effect on level of vitamin C because of inflammation enhancement. Interestingly, vitamin C supplementation seems to be able to prevent and treat the respiratory and systemic infections (35) (Figure 2).

**Figure 3:**
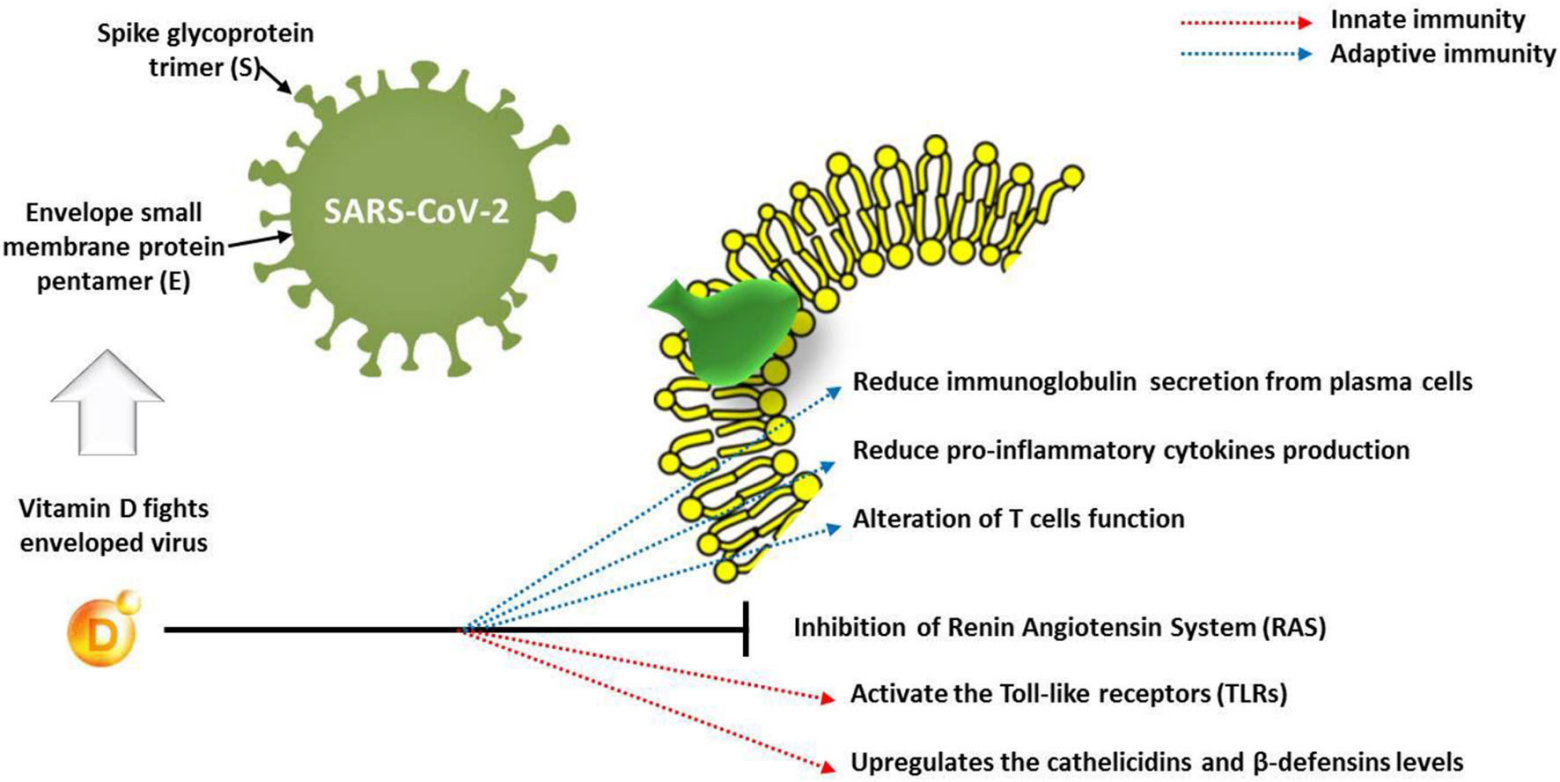
Effect of vitamin D on innate and adaptive immune response in COVID-19 patients.

The function of immune cells is also affected by vitamin D receptors, which indicates that this vitamin deeply influences the immune response to infections. The deficiency of vitamin D, mostly caused by insufficient intake, could result in weak resistance to infections and a higher incidence of various viral diseases. Concerning the COVID-19 pandemic, Ghavideldarestani et al. (36) reported that vitamin D deficiency may have resulted in increased lung injury risk, acute respiratory distress syndrome, diabetes, and cardiovascular symptoms, which are the major risks in COVID-19 patients. The protective role of vitamin D is via enhancement of both innate and adaptive immunity and blocking of the renin–angiotensin system (RAS). Therefore, supplementation of vitamin D could boost the immunity against COVID-19 with subsequent reduction of disease severity in vitamin D-deficient patients. In addition, Panfili et al. (37) reviewed the role of vitamin D supplementation for COVID-19 patients; they concluded that vitamin D is able to interact with innate immune system via activating the Toll-like receptors (TLRs) or upregulating the cathelicidin and β-defensin levels. However, it may interact with adaptive immunity through reduction of immunoglobulin secretion from plasma cells and production of pro-inflammatory cytokines, which alter the function of T cells (Figure 3). The goal should be to raise 25(OH)D concentrations above 40–60 ng/ml (100–150 nmol/L). For treatment of people who become infected with COVID-19, higher vitamin D3 doses might be useful (33).

Concerning the global deficiency of vitamin D, particularly in the Northern Hemisphere populations during the winter, dietary vitamin D supplementation (5,000 IU daily) could have positive outcomes for high-risk groups of individuals (diabetes and obesity), but also during quarantine (38). The occurrence of adverse effects is rare in vitamin D, and modestly high amounts, 2,000–5,000 IU daily, can be consumed for an extended period (39). High amounts of vitamins C and E were found to be ineffective in increasing the immune response, except for vitamin E for viral hepatitis (40, 41). Before the dietary supplementation of vitamin D, it is important to test for possible deficiencies of micronutrients. According to local guidelines, insufficient patients must administer vitamin D at therapeutic doses (39). Other vitamin deficiencies have to be also treated. In patients with infections caused by viruses, the MVM (1 × RDI) supplementation has been suggested (29). In several countries in South Asia, the deficiency of micronutrients, especially vitamin D and B_12_, has been reported (42).

Such defects take place even in high-income countries and are the most common among children and infants, adolescents, the elderly, and during lactation and pregnancy (43). Groups of people with limited diets, such as vegetarians, food allergies, and chronic diseases, are very susceptible to deficiencies of micronutrients (44). To enhance immunity and optimize the nutritional status, it is recommended to consume MVM supplementation daily (29). The immune functions and mechanisms of vitamins are summarized in Table 1.

**Table 1:** The immune functions and mechanisms of vitamins.

**Immune functions and Vitamins mechanisms**	**Vitamins**				
	**Vitamin A**	**Vitamins B6, B12, and folate**	**Vitamin C**	**Vitamin D**	**Vitamin E**
1. Effects on physical and biochemical barriers (Role in maintaining the functional and structural integrities of mucosal cells in innate barriers e.g., gut, skin and respiratory tract).	Important for epithelial tissue to be normally differentiated. Retinoic acid helps in the imprinting of B and T cells and IgA into gut and intestinal tissue, thereby enhancing their immune response. Carotenoids act as immunoregulator via reduction of ROS toxic impacts and regulation of fluidity, and gap junction communications of cell membrane.	Regulate the intestinal immunity and support the gut barriers. B6 mediates the migration of lymphocytes into intestinal tissues. Folate helps the regulatory T cells to survive in the intestinal tract. B12 could be used by gut microbes as a cofactor in metabolic pathways.	Supports the epithelial barriers integrity as it could promote the synthesis of collagen and protect cellular membrane against free radicals. It could enhance the differentiation of keratinocytes, synthesis of lipids, and the migration and proliferation of fibroblasts.	Calcitriol supports gut barriers and regulates defensin and cathelicidin (antimicrobial proteins that could modify the gut microorganisms into healthier compositions). It could protect lungs from infection, upregulate the expressions of tight junction proteins connexion 43 and E-cadherin in the gut, maintain the normal function of renal epithelial barriers, and improve the functions of corneal epithelial barriers function.	Supports the epithelial barriers integrity and protects cellular membranes against free radicals
2. Effects on immune cells. A. Innate immune cells (differentiation, proliferation, functions, and movement).	Regulates the function and numbers of natural killer (NK) cells and the phagocytic activity of macrophage cells.	Maintain or enhance the cytotoxic activity of NK and T cells.	Maintains or enhances the cytotoxic activity of NK and chemotaxis. Helps the movement, function and proliferation of monocytes, neutrophils, and phagocytic cells. Enhances the killing of microbes by enhancing ROS generation and phagocytosis and clear the site of infection from spent neutrophils by macrophages. Decrease tissue damage by attenuating the formation of extracellular trap (NET).	The receptor of vitamin present in monocytes, macrophages, and dendritic cells (DCs). It enhances the monocyte differentiation into macrophages. Calcitriol improves the phagocytic activity of macrophages and promotes their movement.	Maintains or enhances the cytotoxic activity of NK cells. It could provide indirect protection of T cell functions via inhibiting the production of PGE2 from macrophages.
B. Antimicrobial activities.	Inhibits the production of IFN.		Has a role in the production of IFN. Can increase the complement proteins level in serum. High level can possess antimicrobial activity.	Calcitriol can regulate the expression of the antimicrobial proteins defensin and cathelicidin responsible for direct killing of pathogenic bacteria. Inhibits the production of IFN.	
C. Importance in inflammation and antioxidant activities.	Regulates the IL-2 and TNF-α production that are involved in activation of macrophage phagocytic microbial, and oxidative burst activities during inflammatory reactions.	Vitamin B6 help in amino acids synthesis and metabolism and consequently cytokines. Aid in regulation of inflammation process.	Decreases histamine and modulates the production of cytokines. Keeps the cells' redox homeostasis and protects them from ROS. Helps in regeneration of valuable antioxidants like vitamin E and glutathione.	Calcitriol improves the macrophage potential burst activities. Enhances the synthesis of superoxide, decreases the proinflammatory cytokine production from macrophages, and increases the production of anti-inflammatory cytokines.	Acts as antioxidant, protects cells against free radicals. Enhances production IL-2. It could provide indirect protection of T cell functions via inhibiting the production of PGE2 from macrophages.
D. T cell differentiation, proliferation, and normal functions.	Helps the differentiation and development of T helper cells (Th1 and Th2). Aids in the acquisition of mucosal-homing properties by B and T cells.	Vitamin B6 helps the differentiation, maturation, and activities of lymphocytes. Can maintain the immune response of Th1 cells. Vitamin B12 facilitates the T cells' production and regulates the T helper-to-cytotoxic T cell ratio. Folate enhances the immune response mediated by Th1 cells.	Helps the production, differentiation, and proliferation of T cells (especially cytotoxic T cell).	Calcitriol stimulates the innate immunity and inhibits the adaptive immunity via decreasing the proliferation and functions of T cells and antigen-presenting DCs. Help the homing of T cell into the skin.	Helps the proliferation of lymphocytes and enhances the functions and responses of T cells.
3. Antibodies A. Production and development of antibody.	Helps the differentiation and development of T helper cells (Th1 and Th2). Suppresses the production of TNF-α, IL-12, IFN-γ, and Th1 cells and maintains the normal antibody-mediated Th2 responses.	Vitamin B6 helps in amino acids synthesis and metabolism and consequently cytokines and inhibits the Th2 cytokine-mediated activities. Vitamin B12 and folate are involved in production and metabolism of antibodies through folate mechanisms and is important for optimal clonal expansion.	Enhances lymphocyte proliferation, thereby increasing antibody generation.	Calcitriol suppresses the production of antibodies by B cells.	Suppresses the response of Th2.
B. Response to antigens.	Important for normal functions of B cells and helps in the generation of antibody responses to antigens.			Promotes the processing of antigen. Helps in the downregulation of major histocompatibility complex (MHC-II).	Increases the proportions of antigen-experienced memory T cells.
References	(35, 45–51)	(46, 47, 52–56)	(46, 47, 52, 57, 58)	(47, 48, 59–64)	(35, 45–51, 65, 66)

## The Role of Minerals

Minerals are crucial for a potent immune response, and improving the mineral status of the body could be very efficient in boosting the immune system in the defense against the COVID-19 infection (67). The immune functions and mechanisms of minerals are displayed in Table 2. Many studies have proven that many microelements, especially zinc, intensify the immune response (78). Zinc regulates the signaling pathways in the cells of both specific and non-specific immunity (79). Disorders in the homeostasis of zinc alter the immune response in several ways, resulting in abnormal lymphopoiesis, disturbed intercellular cytokine signaling, and weakening the innate immune response through phagocytosis and oxidative burst (80). Zinc deficiency is a very common disorder, and almost 1/5 of the global population is at risk (81). The lack of zinc impedes the immune response, reduces pathogenic resistance, and extends the duration and incidence of pneumonia (82). In the elderly population, dietary supplementation of zinc increases the IL-2 and IL-2R-α mRNA expressions in peripheral blood mononuclear cells (PBMCs) (83). Recent studies showed that zinc affects the polarization of CD4+ T cells on behalf of Th1. In contrast, the up-regulation of IL-12 signaling and transcription factor T-bet activity leads to an increase of IFN-γ (84). Barnett et al. (82) investigated the effects of dietary supplementation with zinc (30 mg/day for 3 months). They reported an increase in zinc concentrations in serum, which was associated with peripheral T cell numbers (85). High intracellular concentrations of zinc with zinc ionophores such as pyrithione can harm the replications of several RNA viruses (86). Concerning the global crisis of the COVID-19 pandemic, the use of zinc as possible supportive treatment in COVID-19 patients could be attributed to its immune enhancement effect and direct antiviral activity (87). In particular, Zn^2+^ cations combined with Zn ionophore pyrithione have a role in inhibition of SARS-coronavirus RNA polymerase (RNA-dependent RNA polymerase, RdRp) activity via reduction of viral replication (86). Interestingly, recent trials concluded the antiviral activity of chloroquine against COVID-19 (88), where they suggested that chloroquine has increased flux of zinc into the cell (89). In continuous context, it was suggested that increased concentration of zinc inside cells by chloroquine could enhance the antiviral effect of zinc against SARS-CoV-2 (90). Similarly, supplementation of zinc without chloroquine may have similar desired effects without the deleterious side effects of chloroquine (91). Another theory for COVID-19 treatment includes targeting Zn ions in the viral protein structure; for instance, in MERS-CoV and SARS-CoV, the antiviral drug disulfiram induced release of zinc from papain-like protease leading to destabilization of viral protein (92). Based on such findings, dietary supplementation with zinc could have positive effects not only on the relief of symptoms related to COVID-19 but also on the virus itself (87).

**Table 2:** The immune functions and mechanisms of minerals.

**Immune functions and mechanisms**	**Minerals**				
	**Iron**	**Zinc**	**Copper**	**Selenium**	**Magnesium**
1. Effects on physical and biochemical barriers (role in maintaining the functional and structural integrities of mucosal cells in innate barriers, e.g., gut, skin, and respiratory tract).	Helps the growth and differentiation of epithelial tissues.	Has a role in maintaining the integrities of mucosal membranes and skin.			
2. Effects on immune cells. A. Innate immune cells (differentiation, proliferation, functions, and movement).	Helps in the bacterial killing by neutrophils through the formation of toxic hydroxyl radicals. It is a component of enzymes essential for immune cell function (such as ribonucleotide reductase helps in synthesis of DNA). Regulates the action and production of cytokines. Iron-rich status negatively regulates. M1 pro-inflammatory response and promotes M2-like macrophage phenotype.	Enhances or maintains the cytotoxic activity of NK cells. Has a key function in the differentiation and growth of immune cells. Enhances the phagocytic activity of peritoneal macrophages and monocytes.	Helps the normal functions of neutrophils, monocytes, NK, and macrophages (copper can accumulate in phagolysosomes in macrophages to counteract certain infections).	Selenoproteins are important antioxidants and help the functions of leukocytes and NK cells.	Acts as cofactor for nucleic acids metabolizing enzymes and helps in replication and repair of DNA, has a role in binding of antigen to macrophage, regulates the activity of leukocyte, and regulates the apoptosis process.
B. Antimicrobial activities.	Helps the production of IFN.	Helps complement activation and has a role in the production of IFN.	Has intrinsic antimicrobial property.	Enhances the production of IFN.	
C. Importance in inflammation and antioxidant activities.	Helps in the production and function of cytokines. Helps in killing of pathogens by neutrophils through generation of ROS.	Acts as anti-inflammatory substance. Modulates the release of cytokines via dampening the pro-inflammatory Th9 and Th17 cell developments. Helps the IL-2, IL-6, and TNF generation. Acts as antioxidant and enhances the activities of antioxidant proteins against ROS and reactive nitrogen species.	Can accumulate at inflammation sites. Acts as a free radical scavenger and enters in the copper/zinc-superoxide dismutase, which is an important enzyme that counteracts the ROS. Helps the IL-2 production and response. Keeps the antioxidant balance inside the cells and has anti-inflammatory action.	Important for the functions of selenoproteins, which are redox regulators and cellular antioxidants against ROS generated during oxidative stresses.	Protects DNA from oxidative damage and decrease the production of superoxide anion.
D. T cell differentiation, proliferation, and normal functions.	Helps in proliferation and differentiation of T cells and regulates the ratio of T helper to T cytotoxic cells.	Promotes cytotoxic T cell proliferation. Helps the production of Th1 cytokines and supports their response. Important for intracellular binding of tyrosine kinase to T cell receptors. Helps the differentiation activation and development of development of Treg cells thereby maintaining immune tolerance.	Helps in proliferation and differentiation of T cells.	Helps in proliferation and differentiation of T cells and improves the count of Th cells.	
3. Antibodies A. Production and development of antibody.		Helps the production of antibodies, mainly IgG.		Has a role in maintaining the levels of antibodies.	Acts as a cofactor in synthesis of antibody helps the antibody-dependent cytolysis and IgM lymphocyte bindings.
B. Response to antigens.		Aids in antibody response and could maintain the immune tolerance (the ability to recognize “self” from “non-self”).			Has an important role in binding of antigens to the RNA of macrophages and helps the antibody-dependent cytolysis.
References	(35, 45, 46)	(1, 35, 45, 47, 65, 66, 68–70)	(46, 47, 54, 70–73)	(45, 47, 54, 74)	(75–77)

Another risk factor that can cause acute infections in the respiratory system is iron deficiency (87). Both hosts and pathogens use iron. Iron deficiency can weaken the immune system, while excessive amounts of iron can cause oxidative stress that promotes mutations of viruses (93). Hemoglobinopathy, hypoxia, and cell iron overload might have a potential additional role. Scientific reports have stated two possible pathophysiological mechanisms: (i) severe acute respiratory syndrome–coronavirus-2 interaction with hemoglobin molecule, through CD26, CD147, and other receptors located on erythrocyte and/or blood cell precursors; (ii) hepcidin-mimetic action of a viral spike protein, inducing ferroportin blockage (94). Selenium has essential roles in the immune response, mostly by incorporating it into selenoproteins (95). Selenium may have a significant place in COVID-19 management, particularly in vulnerable elderly, and might represent a game changer in the global response to COVID-19. Nuclear factor kappaB (NF-kB) signaling pathway plays a role in COVID-19 progression and selenium is a NF-kB inhibitor. Selenium might reduce the effect of SARS-CoV-2 on vascular endothelial cells and aggregation of platelets (96). Supplementing COVID-19-affected patients with selenium might be an efficient way to treat and prevent the novel coronavirus (97). The synergistic use of selenium with saponins from ginseng stem–leaf could activate the immune responses to a live bivalent vaccine of infectious bronchitis coronavirus in chickens (98). The dietary supplementations of selenium could represent an effective method for treating the novel virus of COVID-19 (87). Regarding the aforementioned effects of zinc and selenium, we suggest that dietary supplementation with these two microelements may improve the immune response to viral infections. The suggested dosage for zinc is 20 mg per day while that for selenium is 50 μg per day (4, 11, 99).

Phosphorus (P) is an essential constituent of many important primary cell metabolites such as nucleic acids, phospholipids, lipopolysaccharides, and different cytoplasmic solutes (100–102). P has been suggested in various studies with different species to perform an important role in the immune system and the modulation of P level in the diet could modulate the function and migration of immune cells (100, 101). Similarly, Ca is a normal body constituent and is critical for the immune responses (102).

Calcium phosphate (CaP) in the form of microparticle or nanoparticle has been used as potential adjuvants or vaccine carrier (delivery system) for DNA and peptide vaccines for humans and mammals. CaP nanoparticles have a number of advantages over other inorganic particles. Their biodegradability and biocompatibility are excellent (103); they are native to the body, well-tolerated and absorbed in the body, non-toxic, cost-effective, and easily manufactured and have a high affinity to protein, DNA, antigens, and chemotherapy drugs (104). Calcium phosphate has been used for many years as the adjuvant for diphtheria–tetanus–pertussis (DTP) vaccine and other vaccines (105). CaP nanoparticles have been used to couple several viruses like Epstein–Barr virus and Herpes simplex virus-2, a potent mucosal adjuvant, and induce both mucosal and cell-mediated immunity to nucleotide vaccines in humans and were used in glycoprotein vaccine (106, 107). Additionally, CaP nanoparticles have also been reported to possess better stimulation of both innate and adaptive immunity in mice (108).

## The Role of Probiotics

The utilization of probiotics has been determined to be very beneficial for improving immunity. *Lactobacillus*-based probiotics are recommended for the prevention of influenza-like viral infections (109, 110). Those patients who had been positively tested for COVID-19 have to undergo a validated screening test for malnutrition (e.g., NRS-2002) (111). Probiotics have important roles in the immune response through the regulation of immune cells in the mucosa and epithelial cells of the intestines (112). However, not every type of probiotics exhibits the same effects in the organism, and to obtain the optimal immunostimulatory results, probiotics should be selected thoughtfully based on the clinical state of the disease (113). *Lactobacillus*-based probiotics have been reported to have beneficial effects in viral infection, and they may be used along with proper energy and nutritional intake (109, 114). A small case series from China revealed that some patients with COVID-19 showed microbial dysbiosis with decreased *Lactobacillus* and *Bifidobacterium* (115). Mak et al. (116) stated that the rationale for using probiotics in COVID-19 is derived from indirect evidence. Blind use of conventional probiotics for COVID-19 is not suggested until we have more understanding of the pathogenesis of SARS-CoV-2 and its impact on the gut microbiota. It is likely that a novel and more targeted approach to modulation of the gut microbiota as one of the therapeutic approaches of COVID-19 and its comorbidities will be required.

## The Role of Dietary Guidelines

In addition to hygienic measures and lifestyle behaviors, dietary guidelines are crucial in preventing and treating viral diseases, such as COVID-19. Every patient should follow the dietary recommendations given by the governing bodies (117). The majority of the guidelines state that the diets should predominately consist of fruits, vegetables, and starchy carbohydrates. The diet should consist of meat or a protein-rich equivalent for vegetarians, at least two portions per day (118). Due to the possible economic and logistical difficulties regarding such protein diets, the supplementation with MVM should be mandatory during the pandemic period. In particular, malnourished individuals and those belonging to risk groups should continuously monitor their nutrient status and should be very careful when composing meals (119). It would be advisable to consult a nutritionist regarding the diets, but due to the current socio-economic situation, this could be difficult.

## The Role of Protein-Rich Foods

Protein-rich foods such as meat, milk, egg, bioactive peptides, and others are important for enhancing the immune systems and the body health. Proteins represent the framework of body cells, defense system, hormones, and enzymes that control the functions of the different body systems (120). Proteins are important nutriments, and their chemical structure is composed mainly of nitrogen, hydrogen, carbon, and oxygen. Protein metabolism has been reported to have important functions in formation of both natural and acquired immunities against different infectious agents. The idea that deficiency of proteins could decrease immune functions may be due to the deficiency of essential amino acids connected with the regulation of the immune system (121). Therefore, some amino acids with beneficial immune modulatory effects have been used for fortifying foods such as “immunity regulators” to enhance the immune system functions (122).

Arginine could be act as a vital regulator of immune system when taken with some cofactors like vitamin B. It could stimulate the secretion of growth hormone by pituitary gland, increase T cell production through enlargement of thymus gland, increase the healing activity of the body, and help cancer prevention (123).

Glutamine is an important amino acid that acts as an oxidative fuel for cell proliferation (blood, intestinal, cancer cells, and others) and rapidly replicating cells such as mucosal cells of gastrointestinal tract, colonocytes, enterocytes, macrophages, and lymphocytes. It is a glutathione precursor and could help in regulation of acid–base balance and transportation of nitrogen between body organs. It was found to increase the number of CD8+, CD4+, and T lymphocytes after bone marrow transplantation (124, 125). Other amino acids such as aspartate, glutamate, and arginine cannot be used as substitution for glutamine for supporting the proliferation of lymphocytes (126, 127).

Supplementary nucleotides have been reported to influence a considerable number of immune functions such as enhancing the maturation of T cells and the activity of natural killer cells. Nucleotides could also improve the delayed hypersensitivity in skin, reverse the immunosuppression induced by starvation and malnutrition, increase the resistance against some infections such as *Candida albicans* and *Staphylococcus aureus*, accelerate the immune responses to vaccines, and increase the titers of antibodies (128–130). Additionally, nucleotides could be included in infant formulas to enhance the function of their immune systems (122).

Fish is a rich source of omega-3 fatty acids, such as docosahexaenoic acid (DHA), which is essential for the immune system. Alagawany et al. (131) illustrated that omega-3 fatty acids are important nutritional factors that modulate immune functions. Therefore, it makes sense to consider the use of n-3 PUFAs for clinical management of COVID-19 patients (132). Nutraceuticals in various combinations and forms have been proven to be very useful in the enhancement of the immune system during infections caused by viruses. Several nutraceuticals have immunostimulatory effects, and among them, fish oil, garlic, cranberries, and broccoli are practical and accessible options (133–136). On the contrary, Rogero et al. (137) reported that although the inflammatory resolution improved by eicosapentaenoic acid (EPA) and DHA could contribute to the recovery of patients infected with SARS-CoV-2, omega-3 fatty acid supplementation cannot be recommended before randomized controlled trials are carried out.

The diets of patients under intensive care should also be recommended by a dietician/nutritionist. Besides, to maintain the homeostasis of nutrient intake, some patients will require an oral nutrition supplement (ONS). During viral infections, the resting energy expenditure was elevated by 10%, and due to such circumstances, the energy intake has to be risen by 10% throughout the disease progression (138). Diabetes patients, as members of the risk group, could have severe disease progress and higher mortality (22), and consequently, they should also have specific diet programs and should be monitored by a nutritionist (139). Regarding diabetes patients with COVID-19, Gupta et al. recently demonstrated clinical considerations for such patients (140).

## Phytogenic Feed Additives

Phytogenic feed additives exert their favorable health improving effects owing to their content of active substances such as polyphenols and secondary metabolites. Polyphenols are found in vegetables, grains, fruits, and green and black tea in different parts of the plant (leaves, roots, flowers, seeds, and fruits) to protect the plant immune system against UV radiation and pests (141–151).

Dietary polyphenols have been reported to have various biological activities including polyphenols, which have immunomodulatory, antioxidant, anti-inflammatory, anti-allergic antimicrobial, ant- mutagenic, and detoxification activities (152). Recent studies showed that polyphenols can modulate the immune functions through binding to cellular receptors and altering the cell pathways, thereby regulating the host immune response (153). For instance, curcumin and resveratrol have been evidenced to inhibit the signaling pathway of nuclear factor-kappa β (NF-κβ) and thereby inhibit the release of the proinflammatory cytokine TNF-α (154). Curcumin has been reported to have a role in activation and proliferation of B and T lymphocytes, increase the antibody titer against some viruses, enhance innate immunity in challenged animals, improve the immune response by increasing leucocyte count and phagocytic activates, and stimulate the function of plasma lysozyme and increased immunoglobulin (146, 155, 156). Curcumin could also improve the functions of T and B lymphocytes, monocytes, heterophils, macrophages, and dendritic cells and reduce the inflammatory responses (157).

Another example of polyphenols is resveratrol, which has been reported to have various biological properties such as immunomodulation, antioxidation, hepatoprotection, anti-inflammation, regulation of lipid metabolism, and others (158–160). Resveratrol could strongly inhibit the generation of ROS in mammalian macrophages and neutrophils, suppress the extracellular and intracellular myeloperoxidase (MPO) activity, and reduce the expression of MPO mRNA in neutrophils (161). Moreover, some polyphenols can modulate the expression of some proinflammatory genes and the production of cytokines and the population of immune cells. Dietary polyphenols can alleviate the inflammatory diseases related to field infections via modulating pathogen recognition receptor-mediated signaling pathways (162).

In addition to the immunomodulatory properties of polyphenol compounds, they also showed effective antioxidant properties. Polyphenols could neutralize the free radicals of both reactive oxygen species (ROS) and reactive nitrogen species (RNS), which can damage the chromosomes and consequently modify the encoded amino acid and the associated biological process (163). Zhong and Zhou (164) similarly reported that exogenous antioxidants, including polyphenols, can act as the initial line of defense in the cell that could protect it from oxidative damage caused by excessive production of free radicals. Among all polyphenols, flavonoids such as naringin and naringenin are the most effective in removing generated free radicals and the injuries caused by them (165).

They exhibited robust antioxidant properties in several *in vivo* disease models and were noticed to dramatically prevent the xanthine oxidase activity in *in vitro* studies (166). In general, polyphenols protect the cells from generated free radicals through some action mechanisms such as activation of antioxidant enzymes; scavenging of ROS by participation as electron donor; restriction of hydroxyl radicals (HO·) formation through chelation of transition metals; inhibition of activities of pro-oxidant enzymes such as protein kinase C, xanthine oxidase, and membrane-associated β-nicotin-amide adenine dinucleotide [NAD(P)H] oxidase; alleviation of nitric oxide (NO·) oxidative stress; reduction of α-tocopherol radicals; and enhancing the antioxidant activities of low-molecular antioxidants such as ascorbate and tocopherols by preventing their oxidation (152, 163, 165).

## Animal Coronaviruses

Animal modeling for the newly emerged human coronavirus (2019-nCoV/SARS-CoV-19) is necessary to investigate the virus pathogenicity and to perform the preclinical testing of the possible vaccines and antiviral agents. Factors that have to be considered when selecting/inventing an animal model of human coronaviruses have been reviewed (167). Ideally, the animal model should express the same kind of virus receptors, and the receptors should be distributed in similar anatomical locations to those of humans, such that they get infection via a similar route of transmission as do human beings (168). The modeled animals should allow the virus to replicate and disseminate to the organs and tissues as in the infected human. Moreover, the infected modeled animals should develop clinical features/morbidity (pathophysiology and clinical manifestations, i.e., signs and symptoms) that resemble human disease and the symptom severity should correlate with the viral load and demographic conditions in human (elderly and immunocompromised subjects are vulnerable to severe disease and mortality).

Numerous animal prototypes have been developed for SARS and MERS caused by the respective coronaviruses (166, 169). These include several strains of inbred mice (young vs. aged) and mice with targeted immune defect/specific gene knockout (170, 171), Syrian golden hamsters (172, 173), cats, ferrets (174, 175), and non-human primates including macaques and African green monkeys (176). These models may be used in the study of SARS-CoV-19 infections, an immune correlate of protection, the protective efficacy of vaccines, and the effectiveness of new therapeutics.

Coronavirus spike glycoprotein (S1 domain) binds to the cognate receptor, and the S2 domain mediates viral envelope fusion with the cell membrane for the genome release into cytosol (virus uncoating process). Antibodies targeting the spike glycoprotein of coronaviruses play a critical role in inhibiting viral entry and uncoating. They have clinical potential in conferring passive immunity in the coronavirus-infected individuals.

Currently, several monoclonal antibodies (both fully human molecules or humanized-version) that object the S1 receptor-binding domain (RBD) and non-RBD and S2 regions of coronaviruses have been produced and examined in cell cultures to determine the neutralizing capacity of viruses and animal models as well to assess the efficiency in prophylaxis and post-exposure stage (177). Such antibodies can be utilized as reagents to alleviate the generation of therapeutic drugs/antiviral inhibitors and vaccines.

Many compliant cell lines to human coronaviruses, including the epithelial cells of monkeys (Vero-B4 and LLC-MK2), are being utilized in the neutralization assays [inhibition of virally induced cytopathic effects (CPE) by different antibody concentrations or plaque assays] for determining the levels of neutralizing titers of the antibodies. The lung cells of goats, kidney cells of alpaca, and dromedary umbilical cord cells were identified to be permissive for MERS-CoV (178). Moreover, pseudo-typed viral particles [virus-like particles (VLPs)] that display spike protein on the surface from SARS-CoV were capable for entry to transfected or permissive cells that overexpress the receptors of virus (179–181). The pseudo-typed virions/VLPs with a genome coding for reporting systems, like green fluorescent proteins (GFP) or luciferase could be utilized for quantitative measurement and assessment of inhibitors/monoclonal antibodies that inhibit the cellular entry of the coronaviruses (182). The assays using the pseudo-typed virions/VLPs are safe and can be done in a BSL-2 facility, as they do not involve infectious viruses. A safety attention on the passive immunization using intact antibodies is the possibility of the antibody-based enrichment of the virus replication. Antibody molecules with an altered Fc fragment (cannot be fixed to the Fc receptors on host cells) or without Fc fragment, e.g., human single-chain antibodies (scFv), Fab, or F(ab′)2, should be a harmless alternative.

Data from animal coronavirus vaccine development suggested that systemic cell-mediated or humoral immune reactions induced by parenteral management may not be enough to inhibit the virus invasion in the respiratory system (183). Since the mucosa in the respiratory system is the first target in coronavirus (SARS-, MERS-, and 2019-nCoV/SARS-CoV-19) transmission and infection, the mucosal immunization such as using intranasal vaccine (184) may be a beneficial method for prophylaxis by inducing the immune response in the mucosa. There are severe differences between the molecular mechanisms of mucosal and systemic immunological response; therefore, at the time being, it is rather hard to propose the surrogate indicator for protective ability against the coronavirus diseases. The best surrogate assay for prevention, as well as the herd immunity toward the various coronavirus diseases, warrants investigation.

## Past Experience, Current Situation, and Future Prospective

Immune systems do great work in fighting foreign cells to protect the body from disease. Of course, when a global pandemic strikes the whole world, humans will feel anxious until they conquer it and be healthy. When the COVID-19 epidemic appeared, all sought to find a vaccine to counter this virus. Given a vaccine if provided, immune systems will need to adapt to the COVID-19-infected body (40, 41). Proper nutrition can help in maintaining immune systems as the frontline of defense. Researchers have believed that the deficiency of a particular nutritional element is implicated in the impaired immune responses (6, 11, 185, 186). Nutrition principles based on using some dietary substances such as trace elements, vitamins, probiotics, and nutraceuticals may be helpful in the possible prevention and management of COVID-19 (187). Control of the COVID-19 outbreak and future epidemics requires global efforts among clinicians, immunologists, nutritionists, researchers, veterinarians, and pharmacists (11, 19, 108, 109). Also, public health awareness should be increased about the role of nutrition in eliminating the virus by boosting the immune system. Finally, better understanding the transmission dynamics, incubation period, and replications of COVID-19, along with finding and developing specific vaccines and therapeutics, will pave the way to end this infection soon (188–190).

The lessons learned from this review clarified the common shared scientific knowledge between human and animal nutrition, and that knowledge gained from animal model experiences can be used as a guide in human experiments. This indicates that vitamins such as vitamins C, E, and D and some micro-minerals such as zinc, iron, and selenium and probiotics are candidates to enhance the immunity to viral infections with its specification for each virus.

In conclusion, the strategies of containment have turned out not to be so effective. Such an approach did not stop the spreading of the virus. Social distancing and hygiene measures were an attempt to inhibit the transmission of the virus to give healthcare workers more time to handle the infected patients and “flatten the curve” to lower the peak and meanwhile buy time as researchers all around the world are trying to find efficient therapeutics. Even though the exact intracellular mechanisms of the immunostimulatory effects of nutraceuticals are not entirely explored, a possible beneficial effect is their anti-oxidative and anti-inflammatory activity. This review paper highlighted numerous favorable outcomes of nutraceutical usage in viral infections for both human and animal application, but based on their composition and extraction methods.

## Author Contributions

All authors listed have made a substantial, direct and intellectual contribution to the work, and approved it for publication.

## Conflict of Interest

The authors declare that the research was conducted in the absence of any commercial or financial relationships that could be construed as a potential conflict of interest.
